# Anticoagulation in a Patient With Mechanical Prosthetic Valves and Calcific Uremic Arteriolopathy on Warfarin

**DOI:** 10.7759/cureus.14196

**Published:** 2021-03-30

**Authors:** Megan C Smith, Evan Gleaves, Aniruddha Singh, Muhammad Akbar

**Affiliations:** 1 Cardiology, University of Kentucky, Bowling Green, USA; 2 Internal Medicine, University of Kentucky, Bowling Green, USA

**Keywords:** calcific uremic arteriolopathy, prosthetic heart valve, oral anticoagulation, bridging anticoagulation

## Abstract

Calciphylaxis, or calcific uremic arteriolopathy (CUA), is a rare vascular calcific disease that is most often associated with renal dysfunction and warfarin, particularly end-stage renal disease (ESRD). This condition causes debilitatingly painful skin lesions, oftentimes plaques, throughout areas of cutaneous and subcutaneous adiposity. The progression of these lesions to black eschar with ulceration is the hallmark of CUA. In this report, we present the case of a Caucasian female with a past medical history of nephrogenic systemic fibrosis (NSF), ESRD, and mechanical aortic and mitral valves, anticoagulated with warfarin, who developed CUA. In the setting of mechanical prosthetic valves, vitamin K antagonists (VKA) and aspirin are the only evidence-based antithrombotic therapies. This case presents challenging decision-making when managing anticoagulant therapy in the absence of applicable guidelines.

## Introduction

Calcific uremic arteriolopathy (CUA) is a rare disease with the first known description in 1855, which is associated with renal dysfunction and warfarin, particularly end-stage renal disease (ESRD). CUA has an incidence rate of 3.49 per 1000 patient-years. This is elevated to 6.24 per 1000 patients-years in warfarin users [1]. There is evidence that the cause of increased incidence of calciphylaxis in warfarin users is secondary to the vitamin K-dependent proteins, matrix Gla protein (MGP), and growth arrest-specific gene 6, which are carboxylated in the vasculature by vitamin K and thus inhibited by warfarin [2]. MGP is produced in bone and vascular smooth muscle and prevents vascular calcification. A defect in the MGP gene causes Keutel syndrome, which is associated with extensive soft tissue and vascular calcification [3].

## Case presentation

**Figure 1:**
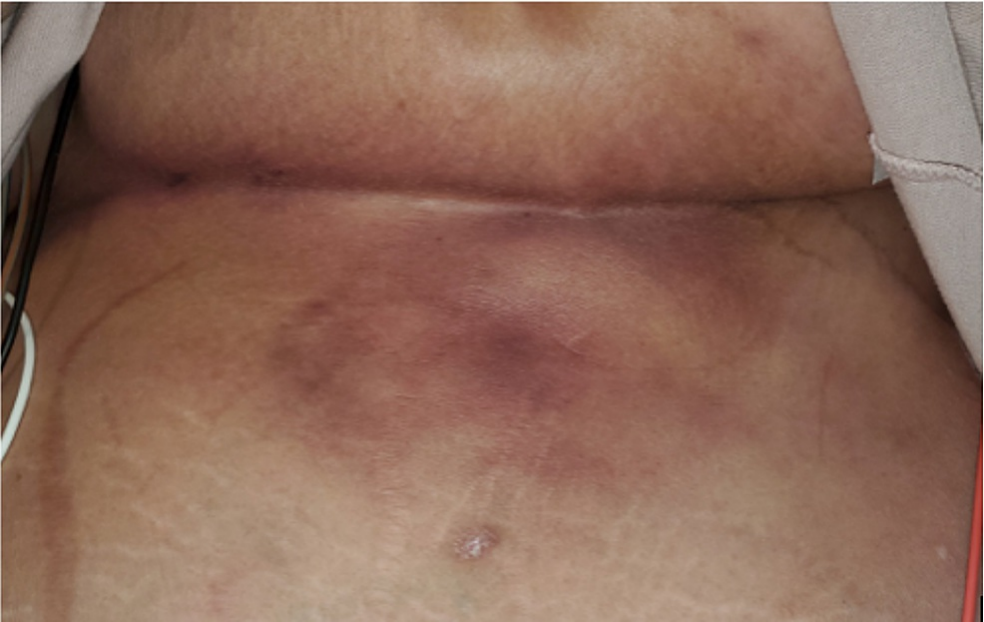
CUA lesion under left breast of patient extending caudally to the abdomen. CUA: calcific uremic arteriolopathy

**Figure 2:**
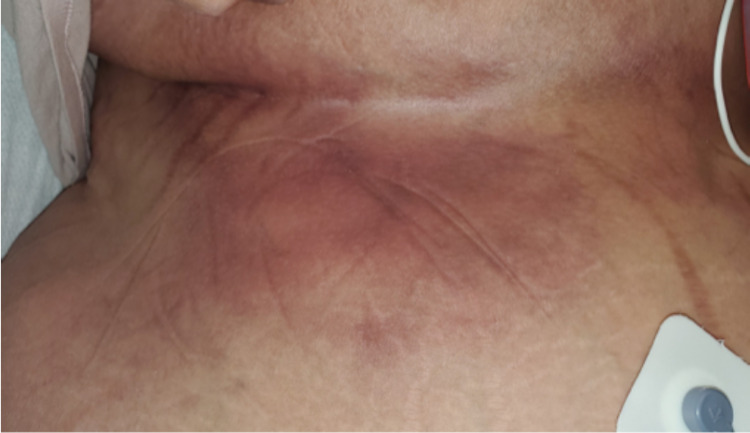
CUA lesion under right breast of patient extending caudally to the abdomen. CUA: calcific uremic arteriolopathy.

The patient is a Caucasian female who presented with the chief complaint of severe burning pain under her breast for roughly one month duration. The pain was located in the right and left upper quadrants. She described it as burning and sensitive to the touch, and “10/10” with palpation. Her skin had become progressively more discolored and tough. On physical exam, there were extensive hard erythematous lesions bilaterally extending from under the breast to the mid abdomen (Figures 1, 2).

Past medical history was significant for ESRD on dialysis for the past 25 years, kidney transplant in 1996 complicated by rejection, nephrogenic systemic fibrosis, 19 mm St. Jude mechanical aortic valve, and 27 mm St. Jude mechanical mitral valve replacements in 2014 due to severe mitral stenosis and moderate aortic stenosis due to extensive calcification, and leadless pacemaker after an atrioventricular (AV) node ablation in 2018. Pertinent medications included warfarin 4 mg daily, which she had been taking for over 10 years.

Transthoracic echocardiogram revealed a preserved ejection fraction of 55-60%, well-seated mechanical prosthetic valves in the aortic and mitral positions with normal peak and mean gradients.

Given her use of warfarin and exquisitely painful skin lesions, warfarin-induced skin necrosis was considered. Her history was significant for a large number of risk factors for CUA, making it a likely explanation. To distinguish between warfarin-induced skin necrosis and CUA, it is important to note the time of onset of warfarin and the response to cessation. Warfarin-induced skin necrosis usually occurs within the first few days of initiation and rapidly clears with cessation. CUA can occur much later and does not resolve quickly with cessation.

Our patient had been taking warfarin for over 10 years, making CUA more likely. Dermatology confirmed the diagnosis of CUA via physical examination and history. She was started on a regimen of sodium thiosulfate 25 g as tolerated during dialysis. Due to her atrial fibrillation and mechanical valves, the continuation of anticoagulation was of paramount importance. Warfarin was held, and she was transitioned to renally dosed enoxaparin to be continued until resolution of CUA with anti-Xa monitoring (target 0.6-1.1 IU/mL). The initial dose was 100 mg of enoxaparin daily (1 mg/kg), however, anti-Xa levels remained subtherapeutic at 0.4-0.5 IU/mL. Enoxaparin dose was subsequently increased to 110 mg daily after three days, resulting in the achievement of therapeutic levels.

On hospital discharge, the painful purpura were improving but not resolved. Sodium thiosulfate 25 g with hemodialysis will continue until the purpura have resolved. Close follow-up with her primary care physician was scheduled soon after hospital discharge with plans to continue enoxaparin and anti-Xa monitoring weekly until CUA resolves.

## Discussion

In the setting of mechanical prosthetic valves, the 2020 updated AHA guidelines for the management of patients with valvular heart disease recommend vitamin K antagonists (VKA) as the only evidence-based therapy for anticoagulation [4]. Given our patient’s diagnosis of CUA and warfarin’s association with the condition, there was an absence of applicable guidelines for anticoagulation while her CUA resolved. The complete discontinuation of her anticoagulation would represent a significant risk given her multiple indications for anticoagulation: atrial fibrillation (CHA2DS2-VASc score of 6), mechanical mitral and aortic valves, and prior stroke. Direct oral anticoagulants have been used with success in patients with CUA [5], however, these agents are also contraindicated in advanced renal failure with the exception of apixaban. Apixaban showed a lower risk of bleeding compared to warfarin in patients with renal disease excluding ESRD [6]. Direct thrombin inhibitors such as dabigatran are indicated as anticoagulation for atrial fibrillation but are contraindicated in patients with mechanical heart valves due to increased risk of thromboembolic and bleeding complications compared to warfarin [7]. There is limited evidence that heparin is a viable substitution for anticoagulation in patients with CUA on warfarin [8]. While there have been no large-scale trials indicating unfractionated heparin or low-molecular-weight heparin (LMWH) for patients with mechanical heart valves, there is a class I recommendation for LMWH when anticoagulation with warfarin must be interrupted [9,10]. In light of this, we believe that our patient was best served by the selection of the LMWH enoxaparin for anticoagulation until warfarin can be continued.

## Conclusions

CUA is a rare disease often associated with ESRD and warfarin. Management decisions are further complicated when the patient has mechanical prosthetic valves. Given the rarity of this scenario, there are no specific guidelines. Extrapolating from the most recent guidelines for interrupting anticoagulation in a patient with mechanical prosthetic valves, we managed this patient with renally dosed enoxaparin until we can consider resuming warfarin again.
